# Fruit, berry, and vegetable consumption and the risk of islet autoimmunity and type 1 diabetes in children—the Type 1 Diabetes Prediction and Prevention birth cohort study

**DOI:** 10.1016/j.ajcnut.2023.12.014

**Published:** 2023-12-23

**Authors:** Markus Mattila, Hanna-Mari Takkinen, Essi J Peltonen, Anna-Leena Vuorinen, Sari Niinistö, Johanna Metsälä, Suvi Ahonen, Mari Åkerlund, Leena Hakola, Jorma Toppari, Jorma Ilonen, Riitta Veijola, Tari Haahtela, Mikael Knip, Suvi M Virtanen

**Affiliations:** 1 Faculty of Social Sciences, Unit of Health Sciences, Tampere University, Tampere, Finland; 2 Tampere University Hospital, Wellbeing Services County of Pirkanmaa, Tampere, Finland; 3 Department of Public Health and Welfare, Finnish Institute for Health and Welfare, Helsinki, Finland; 4 Institute for Molecular Medicine Finland (FIMM), University of Helsinki, Helsinki, Finland; 5 Institute of Biomedicine, Research Centre for Integrative Physiology and Pharmacology, and Centre for Population Health Research, University of Turku, Turku, Finland; 6 Turku University Hospital, Department of Pediatrics, Turku, Finland; 7 Immunogenetics Laboratory, Institute of Biomedicine, University of Turku, Turku, Finland; 8 Department of Pediatrics, PEDEGO Research Unit, Medical Research Center, University of Oulu, Oulu, Finland; 9 Oulu University Hospital, Department of Children and Adolescents, Oulu, Finland; 10 Skin and Allergy Hospital, Helsinki University Hospital, Helsinki, Finland; 11 Research Program for Clinical and Molecular Metabolism, Faculty of Medicine, University of Helsinki, Helsinki, Finland; 12 Tampere University Hospital, Department of Pediatrics, Tampere, Finland; 13 Center for Child Health Research, Tampere University and Tampere University Hospital, Tampere, Finland

**Keywords:** islet autoimmunity, type 1 diabetes, child, cohort study, fruits, vegetables, berries, joint models

## Abstract

**Background:**

Prospective studies investigating the association among fruit, berry, and vegetable consumption and the risk of islet autoimmunity (IA) and type 1 diabetes (T1D) are few.

**Objectives:**

In this cohort study, we explored whether the consumption of fruits, berries, and vegetables is associated with the IA and T1D development in genetically susceptible children.

**Methods:**

Food consumption data in the Finnish Type 1 Diabetes Prediction and Prevention (DIPP) cohort study were available from 5674 children born between September 1996 and September 2004 in the Oulu and Tampere University Hospitals. Diet was assessed with 3-d food records at the age of 3 and 6 mo and annually from 1 to 6 y. The association between food consumption and the risk of IA and T1D was analyzed using joint models adjusted for energy intake, sex, human leukocyte antigen (HLA) genotype, and a family history of diabetes.

**Results:**

During the 6-y follow-up, 247 children (4.4%) developed IA and 94 (1.7%) T1D. Furthermore, 64 of 505 children with at least 1 repeatedly positive autoantibody (12.7%) progressed from islet autoantibody positivity to T1D. The consumption of cruciferous vegetables was associated with decreased risk of IA [hazard ratio (HR): 0.83; 95% credible intervals (CI): 0.72, 0.95, per 1 g/MJ increase in consumption] and the consumption of berries with decreased risk of T1D (0.60; 0.47, 0.89). The consumption of banana was associated with increased risk of IA (1.08; 1.04, 1.12) and T1D (1.11; 1.01, 1.21). Only the association between banana and IA remain significant after multiple testing correction.

**Conclusions:**

In children genetically at risk for T1D, the consumption of cruciferous vegetables was associated with decreased risk of IA and consumption of berries with decreased risk of T1D. In addition, the consumption of banana was associated with increased risk of IA and T1D.

## Introduction

Diet may play role in type 1 diabetes (T1D) development [1,2]. Fruits, berries, and vegetables are known to have beneficial effects on inflammation owing to antioxidants and bioactive compounds, such as flavonoids [3]. Fruits, berries, and vegetables are primary sources of vitamin C, and high plasma vitamin C status has been associated with decreased risk of islet autoimmunity (IA) [4]. Furthermore, the consumption of fruits, berries, and vegetables might promote health by modulating gut microbiota [5] or increasing gut microbial diversity [6] and, thus, potentially protecting against autoimmune diseases, such as T1D. Furthermore, it is suggested that endophytic microbes inside the plant and ectophytic microbes, such as gammaproteobacteria on the surface of fresh fruits, berries, and vegetables might have anti-inflammatory properties [[7], [8], [9]]. However, long storage and processing, such as washing, peeling, and cooking decreases the microbial diversity and, thus, exposure to these microbes particularly in high hygiene urban environments.

Prospective studies assessing the association of the consumption of fruits, berries, and vegetables with the risk of autoimmune diseases, such as T1D are few. In a nested case–control study within the Type 1 Diabetes Prediction and Prevention (DIPP) cohort, the consumption of fruit or berry juices during childhood was associated with increased risk of IA whereas that of solid fruits and vegetables were not [10]. Whether the consumption of processed and fresh/unprocessed fruits, berries, and vegetables differ in relation to the association with IA and T1D has not been previously studied. The consumption of fruits and vegetables has been associated with decreased risk of asthma [11] although prospective longitudinal studies with total diet assessment and information on processing of fruits and vegetables are so far limited to one previous study in DIPP [12], in which only leafy vegetable consumption was weakly associated with reduced risk of asthma and no effect of processing was seen.

The aim of our current prospective study was to investigate whether longitudinal consumption of fruits, berries, and vegetables is associated with the development of IA or T1D in children genetically at risk for T1D. We hypothesized that the consumption of fruits, berries, and vegetables is associated with decreased risk of IA and T1D, and the association is more pronounced for fresh/unprocessed fruits, berries, and vegetables.

## Methods

### Subjects

This study is a part of the DIPP Nutrition Study within the larger DIPP study. DIPP study is an ongoing multidisciplinary prospective population-based birth cohort study screening human leukocyte antigen-DQ beta 1 isotype (HLA-DQB1)-conferred susceptibility to T1D using cord blood samples [13]. Families with newborn infants are recruited from the regions of Oulu, Tampere, and Turku University Hospitals in Finland. Infants carrying a high or moderate genetic risk are invited to a follow-up visit scheduled for 3, 6, 12, 18, and 24 mo and, thereafter, annually ≤15 y of age or at the onset of T1D. The present study included children born between September 1996 and September 2004 in the Oulu and Tampere University Hospitals comprising 6080 children of which 5626 were included in the IA cohort and 5674 were included in the on T1D cohort. The children in the IA cohort were included in the T1D cohort. The inclusion criteria for the IA cohort in the current study were at least 1 autoantibody assessment and at least 1 completed day in a 3-d food record available before the assessment of autoantibodies. The criteria for T1D cohort were available information on T1D status of the child and at least 1 completed food record.

### Outcome variables

Islet cell antibodies (ICAs) were screened at 3-mo to 12-mo intervals [14]. If a participant tested positive for ICA, all available samples from the subject in question were analyzed for insulin autoantibodies, glutamic acid decarboxylase autoantibodies, and islet antigen-2 autoantibodies. IA was defined as repeated positivity for ICA and at least 1 other autoantibody or having T1D. Data of diagnosis of T1D were obtained from the Finnish Pediatric Diabetes Register in May 2017 and the University Hospitals. Children not identified in the diabetes register were considered free from T1D. In progression analyses, risk of T1D was assessed among children who were repeatedly positive for at least 1 autoantibody (ICAs and insulin, glutamic acid decarboxylase, and islet antigen-2 autoantibodies). The children in the progression cohort were included in the IA cohort.

### Genetic methods

HLA-DQ genotyping using panels of sequence-specific oligonucleotide probes has been described previously [13]. The *HLA-DQB1(∗02/∗03:02)* genotype represent “high” and *HLA-DQB1∗03:02/x (x ≠∗02, ∗03:01, ∗06:02)* “moderate” risk for T1D.

### Dietary methods

Childhood diet was assessed with 3-d food records (2 wk and 1 weekend day) at 3, 6, and 12 mo and 2-y, 3-y, 4-y, 5-y, and 6-y visits, as described previously [10]. Written instructions were provided for the families to aid the recording of portion sizes, recipes, preparation methods, and brand names. Information on any special diets or dietary restrictions was inquired with a separate structured questionnaire filled in by the families at each visit. Trained study nurses checked and completed the food records at return and inquired any potential missing items [10]. Trained nutrition researchers prepared the food records for calculation. The dietary data calculation was made using in-house software (Finessi) of the Finnish Institute for Health and Welfare, Finland, based on the Finnish national food composition database (Fineli) [15]. For breastfed children, we estimated the total energy intake based on age, bodyweight, and the expected energy deposition needed for growth [16]. We wanted to take into account age-related and sex-related differences in energy intake and fruit/vegetable consumption, and thus, we adjusted the outcome analyses for total energy intake.

Our existing database and software (Fineli and Finessi) do not enable us to calculate unprocessed (raw) and processed (heat treated) fruits, berries, and vegetables separately owing to the structure of the calculation process. Thus, we created a new external calculation model for this purpose [12]. Fruits, berries, and vegetables were determined as processed if they were at some point during preparation heated (including pickled and dried fruits, berries, and vegetables). Fruits, berries, and vegetables were classified as fresh/unprocessed if they were not heated at any point of their processing. Some minor sources of fruits, berries, and vegetables were excluded from the calculation model, for example, dried herbs and spices, fruits and berries in flavored yogurts, and potato chips, because of their low relevance as a source of fruits and/or vegetables in the childhood diet.

**FIGURE 1:**
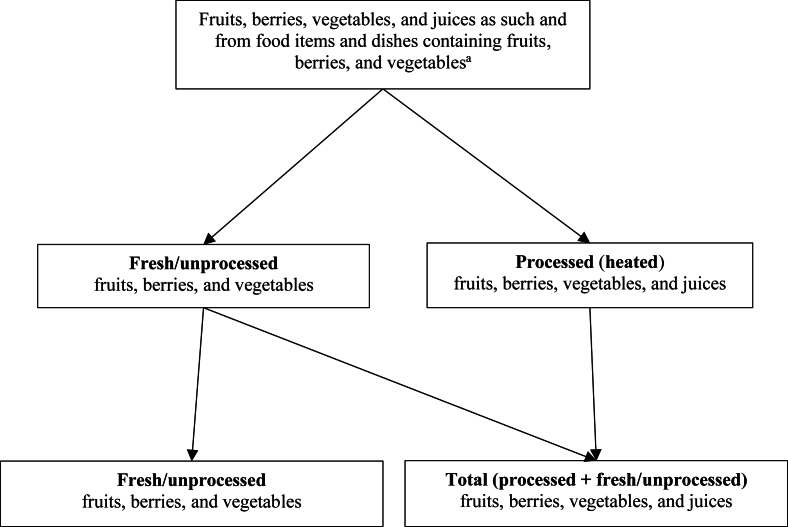
Processing-based classification of fruits, berries, vegetables, and juices. ^a^Some food items excluded from the classification owing to low relevance as source of vegetables and fruits, for example, dried herbs and spices, fruits and berries in flavored yogurts, and potato chips.

The classification of unprocessed and processed fruits, berries, and vegetables used in these analyses are presented in Figure 1. In the outcome analyses, we studied consumption of individual fruits, berries, and vegetables in the 2 following consumption categories: *1*) total consumption that contained both unprocessed and processed fruits, berries, and vegetables and *2*) unprocessed fruits, berries, and vegetables. Consumption of potato and fruit juices were included only in the processed category as all food items in these groups were classified as heated. Frozen vegetables, if their frozen origin was known, were categorized as processed as they are cooked or blanched before freezing. Frozen fruits and berries are typically frozen as such and, thus, categorized as fresh/unprocessed. In the analyses described in this article, the consumption of fresh/unprocessed and processed citrus fruits, grape, melons, exotic fruits, mushrooms, nuts, seeds, and seaweed were not analyzed separately as only a small proportion (<20%–30%) of the children consumed them but they were included in the assessment of total fruits and vegetables. The fruit and vegetable groups assessed in the present study and examples of food items within the groups are presented in Table 1.

**TABLE 1:** Fruit and vegetable groups included in this study and examples of food items.

Main group	Subgroup	Examples of included fruit or vegetable
Fruits (without berries)^1^	Apple fruits	Apple, pear
	Banana	
	Citrus fruits^2^	
	Other fruits^2^	Grape, melons, exotic fruits
	Berries	Strawberries, bilberries
Vegetables (without potato)	Cruciferous vegetables	White cabbage, cauliflower, broccoli
	Fruit vegetables	Tomato, cucumber
	Leafy vegetables	Lettuce, spinach
	Legumes	Beans, peas
	Onions	Onions, leeks
	Root vegetables	Carrot, rutabaga, beet
	Mushrooms^2^	
	Nuts and seeds^2^	
	Potato	Boiled potatoes, French fries, and other potato-based products
Juices	100% fruit or vegetable juices	Apple juice, orange juice, carrot juice

1 Originally, the analyses were made for fruits as a whole group (berries analyzed separately). Because an association was detected for the whole group of fruits, this group was divided into subgroups: apple fruits, banana, citrus fruits, and other fruits. Banana was analyzed as an own group because it is commonly used by Finnish children.

2 Not analyzed separately owing to small percentage (<20%–30%) of users but included in the assessment of total fruits and vegetables.

### Sociodemographic characteristics

Information on diabetes status (all types of diabetes) of the ﬁrst-degree relatives (yes/no), child’s sex (male/female), and mother’s vocational education (none, vocational, secondary vocational, university studies, or degree) were collected from parents after delivery using a structured questionnaire. Weight and length were assessed at study visits and weight-for length *z*-scores were calculated for each visit and sex.

### Ethical aspects

The study adheres to the tenets of the Declaration of Helsinki, and the local ethics committees approved the study protocol. Families gave their written informed consent for the genetic testing of the newborn infant and for their participation in the follow-up study.

### Statistical methods

Joint models (JMs) that combine longitudinal and survival data into a single model [17] were used to analyze the association between the longitudinal consumption of fruits, berries, and vegetables and the development of IA and T1D in children. The JM enables the reconstruction of a complete exposure profile for each child even if the series of repeated dietary measurements are incomplete. Children with more frequently observed dietary data contributed more to the analysis. In comparison with traditionally used time-dependent Cox regression model with continuous longitudinal food consumption measurements used as a piecewise constant step function, JM provides more accurate utilization of food consumption measurements [18,19]. The longitudinal submodel of the JM was built through linear mixed-effect (LME) models, which can consider repeated measurements through random (subject-specific) effects. A diagonal structure was assumed for the variance–covariance matrix of the random effects. Food consumption from 3 mo (IA and T1D) or first seroconversion (progression), up to detected outcome or 6 y of age was modeled using piecewise natural cubic spline functions with 3 knots in the LME submodels. The locations of knots were defined by use of an algorithm that selected the best suitable combination of knots by fitting all relevant combinations and selecting the best fitting model based on the Bayesian information criterion. The algorithm required that there had to be at least 2 recording points (or 3 mo follow-up time for progression) before, or at the first knot, and after, or at the last knot, and 1 y between the knots. In progression analyses, if fruit, berry, or vegetable consumption measurement prior to the first seroconversion was available for an individual, a linear interpolant between that and the following measurement (the first one after time scale origin) was fitted, and the consumption at the first seroconversion was approximated from the interpolation line. This was done to ensure an appropriate fit for the individual LME curves before the first available measurement after the time origin, since the ages, and thus the consumption, at the first seroconversion varied a lot between individuals, and no one had consumption measured exactly at that point.

The survival submodel was built using the structure of the Cox proportional hazards model. The children were followed from 3 mo of age (IA and T1D), or from the first seroconversion (progression), up until the date of IA detection/T1D diagnosis, or the age of 6 y, whichever came first. The baseline hazard was set as a piecewise constant with jump points at the ages/times of 1.99 and 3.99 y, given the maximum follow-up of 5.75 y.

The JMs were estimated within a Bayesian framework by using Markov chain Monte Carlo (MCMC) algorithms. The parameter values from the submodels were given as prior information for the full posterior distribution. Three chains were set for the MCMC and the Gelman-Rubin diagnostic was used to check the similarity of the obtained 3 parameter distributions and, thus, assesses the convergence of the MCMC sampler [20]. The diagnostic values <1.1 were considered as convergence.

The JMs were analyzed separately for each vegetable and fruit group variable with energy adjustment for all outcomes and for IA also without energy adjustment. Multivariate nutrient density method was used in the energy adjustment [21]: the consumed amount of food (in grams) was divided by the total energy intake (in megajoules), and the received variable was included in a model as a covariate together with the total energy intake. All models were adjusted for variables previously observed to be potential confounders; sex (male or female), HLA genotype (high or moderate risk), and a family history of diabetes of any type (yes or no). Progression analyses were also adjusted for age at seroconversion. The models provided the posterior mean estimates as hazard ratios (HRs) and 95% credible intervals (CIs). A current value association structure was used, and thus, the HR at a given point in time *t* is provided for a 1-unit [10 g or 1 g/megajoule (MJ)] increase in the longitudinal value of the vegetable or fruit consumption at the same time point *t*. Multiple testing was controlled for using the false discovery rate method (a step-up procedure using a 0.05 level as the criterion).

Higher level of maternal education was associated with decreased risk of IA and increased risk of progression from IA to T1D in our previous study [22]. Thus, we performed additional analysis further adjusting for maternal education. Further, we performed an additional outcome analysis adjusting for weight-for-length *z*-score to assess whether growth/overweight confounds our results. To assess whether avoidance of certain foods could affect the observed association among fruit, berry, and vegetable consumption and the risk of outcomes, we performed a sensitivity analysis where we excluded children avoiding fruits, vegetables, or major food allergens: dairy or cereals. Because the use of berries and cruciferous vegetables were low, we wanted to know, whether the association between consumption of them on the risk T1D development differed between consumers and nonconsumers. Thus, we performed additional sensitivity analyses using berry and cruciferous vegetable consumption as a categorical variable. We included the 2-group classification variable in an adjusted Cox regression model to investigate the IA/T1D risk depending on the fruit or vegetable consumption status (no/yes) with the limit of 1 g at any age point. Missing information in the family history of diabetes variable was treated as a defined category to retain a maximum number of subjects in the analyses. The analyses were conducted using the JM function from the JMbayes2 package [23] in R version 4.2.1.

## Results

**FIGURE 2:**
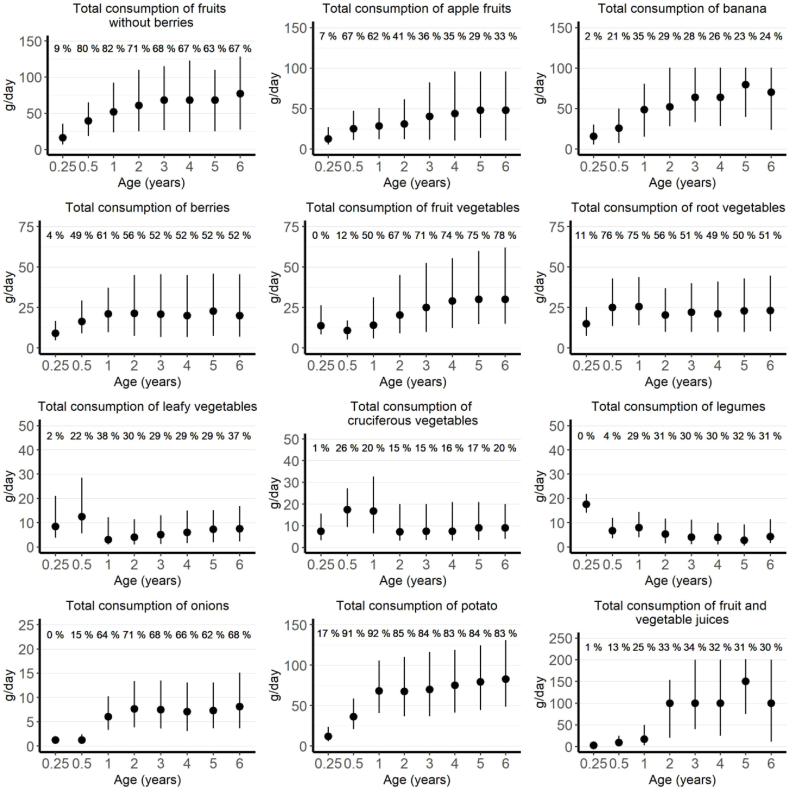
Median consumption and proportion of users of fruits, berries, and vegetables by age among users of each respective food/food group with interquartile ranges by age in 5674 children in the DIPP study. DIPP, Type 1 Diabetes Prediction and Prevention Study.

**FIGURE 3:**
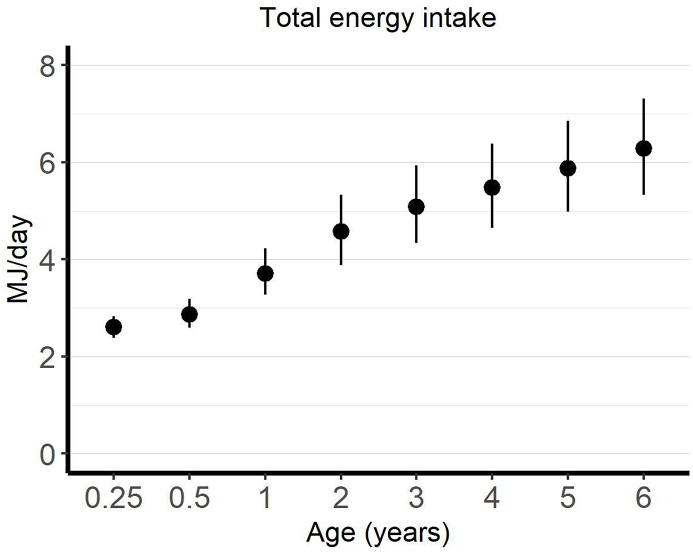
Mean (SD) intake of energy (MJ/d) by age in all 5674 children in the DIPP study. DIPP, Type 1 Diabetes Prediction and Prevention Study.

The distribution of children in relation to outcome and background variables are presented in Table 2. The participant flow chart is presented in Supplemental Figure 1. Of the 6080 participants enrolled for the follow-up, the food record data were available from 5626 children (93%) in the IA cohort and 5674 (93%) in the T1D cohort. From the 5626 children in the IA cohort, 247 children (4.4%) developed IA during the 6-y follow-up. Of the 5674 children in the T1D cohort, 94 children (1.7%) developed T1D during the 6-y follow-up. A total of 505 children developed repeated positivity to at least 1 autoantibody of which 64 (12.7%) developed T1D by the age of 6 y after first seroconversion. The dropout rates among the 5626 participants were for serum autoantibody measurements at 1-y, 2-y, and 6-y follow-up 6%, 14%, and 35% and for food records 15%, 25%, and 55%, respectively. The total number of food record days was 81,075 in 5674 children aged 3 mo to 6 y, and the average food record days per child was 14.3. A total of 25 children had only 1 food record day available, whereas 26 children had only 2 food record days in one of the visits. Median consumption and proportion of users of fruits, berries, and vegetables by age among users of each respective food/food group is presented in Figure 2. Mean intake of energy (in MJ per day) among all 5674 DIPP children is presented in Figure 3.

**TABLE 2:** Characteristics of children in relation to islet autoimmunity, type 1 diabetes, and progression to type 1 diabetes^1^

Characteristic	Islet autoimmunity cohort		Type 1 diabetes cohort^2^		Progression to type 1 diabetes cohort^2^	
	Total (*n* = 5626)	Islet autoimmunity (*n* = 247)	Total (*n* = 5674)	Type 1 diabetes (*n* = 94)	Total (*n* = 505)	Type 1 diabetes (*n* = 64)
Sex						
Male	2988 (53.1)	148 (59.9)	3010 (53.0)	54 (57.4)	285 (56.4)	42 (65.5)
Female	2638 (46.9)	99 (40.1)	2664 (47.0)	40 (42.6)	220 (43.6)	22 (34.4)
HLA-DQB1–conferred risk^3^						
High	1102 (19.6)	77 (31.2)	1109 (19.5)	35 (37.2)	116 (23.0)	23 (35.9)
Moderate	4524 (80.4)	170 (68.8)	4565 (80.5)	59 (62.8)	389 (77.0)	41 (64.1)
Family history of diabetes^4^						
Yes	333 (5.9)	30 (12.2)	333 (5.9)	14 (14.9)	43 (8.5)	9 (14.1)
No	5080 (90.3)	211 (85.4)	5125 (90.3)	78 (83.0)	452 (89.5)	54 (84.4)
Missing	213 (3.8)	6 (2.4)	216 (3.8)	2 (2.1)	10 (2.0)	1 (1.6)
Maternal vocational education						
None	356 (6.3)	26 (10.5)	360 (6.3)	8 (8.5)	35 (6.9)	3 (4.7)
Vocational school or course	1499 (26.6)	61 (24.7)	1510 (26.6)	21 (22.3)	120 (23.8)	12 (18.8)
Secondary vocational education	2395 (42.6)	88 (35.6)	2412 (42.5)	34 (36.2)	213 (42.2)	23 (35.9)
University studies or degree	1220 (21.7)	65 (26.3)	1234 (21.7)	28 (29.8)	128 (25.3)	24 (37.5)
Missing	156 (2.8)	7 (2.8)	158 (2.8)	3 (3.2)	9 (1.8)	2 (3.1)
Median age at seroconversion (IQR)		2.5 (1.3–3.6)		4.0 (2.9–5.0)	1.9 (1.2–3.5)	3.5 (2.4–4.7)

1 Figures are number of children and percentage in parenthesis unless otherwise stated.

2 Of the 94 T1D children, 14 did not meet the criteria of repeated positivity to at least 1 autoantibody and 15 were excluded owing to availability of food records only before seroconversion. Furthermore, 1 child did not have any measurements on autoantibodies. This results in 64 children who developed T1D by the age of 6 y after first seroconversion.

3 High-risk genotype: human leukocyte antigen-DQ β 1 isotype (*HLA-DQB1)(∗02/∗03:02)*. Moderate-risk genotype *HLA-DQB1∗03:02/x (x ≠∗02, ∗03:01, ∗06:02)*.

4 All types of diabetes.

When adjusted for sex, HLA genotype, and a family history of diabetes, the total consumption (fresh/unprocessed + processed) of cruciferous vegetables was associated with decreased risk of IA without energy adjustment (HR: 0.63; 95% CI: 0.31, 0.97, per 10-g increase in consumption) or with energy adjustment (HR: 0.83; 95% CI: 0.72, 0.95, per 1-g/MJ increase in consumption) (Table 3). The absolute total consumption (per 10-g increase) of fruits (HR: 1.05; 95% CI: 1.01, 1.09), banana (HR: 1.14; 95% CI: 1.05, 1.23), and potato (HR: 1.06; 95% CI: 1.01, 1.11) was associated with increased risk of IA but after the energy adjustment (per 1-g/MJ increase), the association was significant only for the consumption of fruits (HR: 1.02; 95% CI: 1.00, 1.04) and banana (HR: 1.08; 95% CI: 1.04, 1.12). Further adjustment for maternal education did not change the results (data not shown). The consumption of fresh/unprocessed fruits and banana was associated with increased risk of IA with or without energy adjustment (Supplemental Table 1). The consumption of fresh/unprocessed berries was associated with decreased risk of IA but only without energy adjustment.

**TABLE 3:** Associations of consumption of fruits, berries, and vegetables (total) with the risk of islet autoimmunity, type 1 diabetes, and progression to type 1 diabetes from joint modeling

	Islet autoimmunity				Type 1 diabetes		Progression to type 1 diabetes	
	HR (95% CI)^1^*n* = 5626 (cases, *n* = 247)	*P*	Energy-adjusted HR (95% CI)^2^*n* = 5626 (cases, *n* = 247)	*P*	Energy-adjusted HR (95% CI)^2^*n* = 5674 (cases, *n* = 94)	*P*	Energy-adjusted HR (95% CI)^3^*n* = 505 (cases, *n* = 64)	*P*
Fruits (without berries)	1.05 (1.01, 1.09)	0.018	1.02 (1.00, 1.04)	0.020	1.04 (1.00, 1.08)	0.036	1.05 (1.00, 1.09)	0.049
Apple fruits	1.07 (0.98, 1.16)	0.132	1.02 (0.98, 1.06)	0.336	1.07 (0.99, 1.14)	0.081	1.11 (1.00, 1.23)	0.043
Banana	1.14 (1.05, 1.23)	0.005	1.08 (1.04, 1.12)	<0.001^4^	1.11 (1.01, 1.21)	0.031	1.08 (0.98, 1.18)	0.116
Berries	0.93 (0.82, 1.06)	0.325	0.97 (0.90, 1.03)	0.342	0.60 (0.47, 0.89)	0.002	0.94 (0.77, 1.08)	0.449
Fruit vegetables	1.03 (0.96, 1.10)	0.362	1.02 (0.98, 1.05)	0.388	1.05 (0.98, 1.10)	0.131	1.03 (0.95, 1.10)	0.399
Root vegetables	1.00 (0.87, 1.12)	0.963	0.98 (0.93, 1.03)	0.445	0.91 (0.79, 1.04)	0.185	0.96 (0.74, 1.20)	0.784
Leafy vegetables	0.91 (0.65, 1.20)	0.578	0.94 (0.83, 1.04)	0.252	0.90 (0.72, 1.11)	0.431	1.03 (0.75, 1.28)	0.730
Cruciferous vegetables	0.63 (0.31, 0.97)	0.036	0.83 (0.72, 0.95)	0.007	0.85 (0.61, 1.08)	0.301	0.81 (0.46, 1.18)	0.376
Legumes	0.80 (0.39, 1.33)	0.506	0.98 (0.73, 1.22)	0.976	.^5^	—	0.69 (0.24, 1.53)	0.467
Onions	1.23 (0.81, 1.83)	0.352	1.20 (0.98, 1.44)	0.077	1.52 (1.00, 2.17)	0.051	1.41 (0.74, 2.54)	0.267
Potato	1.06 (1.01, 1.11)	0.009	1.02 (1.00, 1.04)	0.051	1.03 (0.98, 1.07)	0.262	1.08 (1.03, 1.14)	0.004
Fruit and vegetable juices	1.00 (0.97, 1.03)	0.980	1.00 (0.99, 1.01)	0.741	1.00 (0.97, 1.02)	0.830	1.01 (0.97, 1.04)	0.578

1 Hazard ratios (HRs) and credible intervals (CIs) per consumption of 10 g of food item: adjusted for sex, HLA genotype, and family history of diabetes of any type.

2 HRs and CIs per consumption of 1 g/MJ of food item: adjusted for sex, HLA genotype, family history of diabetes of any type, and total energy intake.

3 HRs and CIs per consumption of 1 g/MJ of food item: adjusted for, energy intake, sex, HLA genotype, family history of diabetes of any type, and age at seroconversion.

4 *P* < 0.05 after multiple testing correction.

5 Not assessed owing to convergence problems.

The energy-adjusted (per 1-g/MJ increase) total consumption of berries was associated with decreased risk of T1D (HR: 0.60; 95% CI: 0.47, 0.89) whereas the total consumption of fruits (HR: 1.04; 95% CI: 1.00, 1.08) and banana (HR: 1.11; 95% CI: 1.01, 1.21) was associated with an increased risk of T1D (Table 3). Further adjustment for maternal education did not change the results (data not shown). The energy-adjusted consumption of fresh/unprocessed fruits, banana, and apple fruits was associated with increased risk of T1D (Supplemental Table 1).

The energy-adjusted (per 1-g/MJ increase) total consumption of fruits (HR: 1.05; 95% CI: 1.00, 1.09), apple fruits (HR: 1.11; 95% CI: 1.00, 1.23), and potato (HR: 1.08; 95% CI: 1.03, 1.14) was associated with an increased risk of progression to T1D (Table 3). When further adjusted for maternal education, the association was no longer associated at a level of *P* < 0.05 for the consumption of fruits (HR: 1.04; 95% CI: 0.99, 1.09) and apple fruits (HR: 1.08; 95% CI: 0.97, 1.20). The energy-adjusted consumption of fresh/unprocessed fruits and apple fruits was associated with increased risk of progression to T1D (Supplemental Table 1).

The results remained similar when further adjusted for weight-for-length *z*-score (data not shown). Furthermore, the results were similar for all outcomes in the sensitivity analyses where we excluded children avoiding vegetables, fruits, and major food allergens: dairy or cereals (data not shown). When the outcome analyses were corrected for multiple testing, the associations were significant only between consumption of banana and the risk IA (Table 3). In additional sensitivity analyses using categorical consumption variables, the total consumption of berries (consumers compared with nonconsumers—HR: 0.35; 95% CI: 0.24, 0.51) and cruciferous vegetables (HR: 0.50; 95% CI: 0.38, 0.64) was associated with decreased risk of IA but not with risk of T1D (berries—HR: 0.83; 95% CI: 0.45, 1.52; and cruciferous vegetables—HR: 0.86; 95% CI: 0.56, 1.31).

## Discussion

In our large prospective birth cohort of children genetically at risk for T1D, the total consumption of cruciferous vegetables was associated with decreased risk of IA and that of berries with decreased risk of T1D. Conversely, the consumption of banana was associated with increased risk of IA and T1D. The associations of the fresh/unprocessed vegetables and fruits with the T1D outcomes were very similar to those of the total groups including both processed and unprocessed foods.

Consumption of cruciferous vegetables was associated with decreased risk of IA and consumption of berries was associated with decreased risk of T1D. This may result from several mechanisms such as anti-inflammatory properties of polyphenols in fruits, berries, and vegetables, which boost the antioxidant activity [24]. Furthermore, berry consumption may also promote glucose metabolism, improve insulin sensitivity, or modulate gut microbiota [5,25]. We observed in TEDDY study that high plasma ascorbic acid (vitamin C) status might protect against IA [4]. Vitamin C is one of the most prominent antioxidants found in fruits, berries, and vegetables. Cruciferous vegetables are Brassicaceae family vegetables containing several compounds with antioxidant properties such as tocopherols, ascorbic acid, carotenoids, phenolics, and glucosinolates [26]. Glucosinolates in brassica family vegetables have been of interest owing to their potential anti-inflammatory properties [27]. Glucosinolates are also suggested to function via gut microbiota metabolism [28]. Furthermore, berries are prominent source of polyphenols with anti-inflammatory properties possibly via modulation of gut microbiota [5]. Finally, the naturally occurring microbes, especially gammaproteobacteria in fresh/unprocessed fruits, berries, and vegetables, are implicated to have anti-inflammatory properties [29], and they are also suggested to increase gut microbial biodiversity [6]. However, we did not observe substantial differences on the risk of T1D-related outcomes between fresh/unprocessed and processed vegetable, fruit, or berry consumption.

We observed that high consumption of banana was associated with increased risk of IA and T1D and the consumption of apple fruits was associated with increased risk of progression to T1D. Although plant-based foods have several compounds with health benefits, they can also contain detrimental compounds such as pesticides and microbial toxins, which are suggested to increase the risk of T1D development [30,31]. Pesticide residues can be found especially in fruits and vegetables exported from countries outside European Union [32] and exposure to pesticide residue may vary according to growing methods (eg, organic compared with nonorganic). In Finnish children, banana and apple are significant source of carbamate, organophosphate, and azole residues although the exposure does not exceed the acceptable levels [32]. However, studies exploring the association between exposure to pesticides and the risk of T1D are rather limited [31]. We could not assess fruit and vegetable consumption by expected pesticide residual exposure as our database does not include the growing method or country of origin. The association between consumption of apple fruits and progression to T1D was similar but weaker when further adjusted for maternal education. In previous DIPP study, we observed that that higher levels of maternal education were associated with increased risk of progressing from IA to T1D [22]. Thus, our current observation on the association between apple fruit consumption and progression to T1D could be confounded by maternal education or the association was weaker owing to reduced power. The consumption of potato was associated with increased risk of progression from IA to T1D even when adjusted for maternal education. Potato is one of the first solid foods in the diet of Finnish infants, and it consists primarily of quickly digestible starch. Hence, potato has high glycemic index, particularly in pureed form [33]. Previous DAISY study reported that consumption of foods with high glycemic index was associated with rapid progression to T1D in children with IA, which the researchers suggested was due to increased demand of insulin release [34]. Furthermore, higher consumption of carbohydrates and, specifically, sugars was associated with rapid progression to T1D, but starch consumption was not assessed in the DAISY study [35]. Potato and banana have higher carbohydrate content than other fruits and vegetables because of higher starch or sugar content [36]. A similar association has been observed in consumption of other high carbohydrate foods such as cereals and juices [10,37]. Finally, *Streptomyces* sp. toxins found in potatoes have been suggested to contribute to T1D development [30], but human studies are few.

It should be noted that the daily of consumption of fruits, berries, and vegetables in our study was low in comparison with Finnish recommendation for children of ∼250 g/d, which is half of the recommended serving for adults [38].

The strengths of our study include a large study population, longitudinally assessed total diet, regularly maintained food composition database, and a regular assessment of autoantibodies. Our study included accurate recording of food consumption with novel calculation model of fruit, berry, and vegetable consumption, which enabled us to explore the use of fresh/unprocessed and processed fruits and vegetables very accurately. Furthermore, we have previously observed that the 3-day food record used in our study is a valid tool for the assessment of root vegetable consumption and carotenoid intake [39]. To our knowledge, this is the first prospective study to explore the association between longitudinal fruit, berry, and vegetable consumption and risk of IA, T1D, or progression from islet autoantibody positivity to T1D. In addition, whether the consumption of fresh/unprocessed fruits, berries, and vegetables compared with processed ones have different impact on the T1D outcomes is not previously studied. Another strength in our study was the use of joint modeling, which analyses all individual food record days available and includes child-specific consumption trajectories into the models and includes all children regardless of missing data on some of food record days. This decreases the risk of bias owing to missing data. A limitation is that our study cannot determine whether the observed association is causal. Because our study included children genetically at risk of T1D, we cannot generalize results to all children. Our study was observational with growing children, and thus, we cannot exclude the chance of residual confounding owing to unmeasured confounders not being taken into account in this study. Furthermore, after multiple testing correction, the associations were significant only between consumption of banana and the risk IA. Finally, our results are novel, and thus, it is possible that our observations might result by chance or they might reflect specific dietary habits.

In this prospective study with children genetically at risk for T1D, the consumption of cruciferous vegetables was associated with decreased risk of IA and consumption of berries with decreased risk of T1D. In addition, the consumption of banana was associated with increased risk of IA and T1D.

### Author contributions

The author's responsibilities were as follows – MM, A-LV, SN, JM, TH, SMV: designed the study; H-MT, EJP, A-LV: designed and conducted the statistical analyzes; SA, MÅ: designed the calculation model for processed/unprocessed fruits and vegetables and contributed to food composition database work; MM: drafted the manuscript; MM, SN, LH, SMV: interpreted the results; SA: supervised dietary data collection, processing, and analyzes; JI, JT, MK: conceived and designed the DIPP study; MK, RV: were responsible for the autoantibody analyses; and all authors participated in drafting or revising of the manuscript and approved the final version to be published.

### Conflict of interest

The authors report no conflicts of interest.

### Funding

This study was supported by the Research Council of Finland (grants 63672, 68292, 79685, 79686, 80846, 114666, 126813, 129492, 139391, 201988, 210632, 250114, 276475, 308066, and 339922); European Foundation for the Study of Diabetes; Doctoral Programs in Public Health; the Finnish Diabetes Research Foundation; the Juho Vainio Foundation; the Yrjö Jahnsson Foundation; Competitive Research Funding of the Turku and Oulu University Hospitals; the Competitive State Research Financing of the Expert Responsibility area of Tampere University Hospital (grants 9E082, 9F089, 9G087, 9H092, 9J147, 9K149, 9L042, 9L117, 9M036, 9M114, 9N086, 9P057, 9R055, 9S074, 9T072, 9U065, 9V072, 9X062, 9AA084, and 9AB083); the Juvenile Diabetes Research Foundation (grants 4-1998-274, 4-1999-731, 4-2001-435, 1-SRA-2016-342-M-R, 1-SRA-2019-732-M-B, and 3-SRA-2020-955-S-B); the Novo Nordisk Foundation; and the European Union Biomed 2 Program (BMH4-CT98-3314).

### Data availability

Data described in the manuscript, codebook, and analytic code will be made available on request pending (eg, application and approval, payment, and other).

## Appendix A Supplementary data

Multimedia component1 Multimedia component1

Multimedia component2 Multimedia component2
