# Supplementary material for: Fruit, berry, and vegetable consumption and the risk of islet autoimmunity and type 1 diabetes in children—the Type 1 Diabetes Prediction and Prevention birth cohort study
Source: Am J Clin Nutr. 2023 Dec 23;119(2):537–45. doi: 10.1016/j.ajcnut.2023.12.014 (PMC10884602; doi:10.1016/j.ajcnut.2023.12.014)
Supplement: Multimedia component1 [file mmc1.pdf]

Fruit, berry, and vegetable consumption and the risk of islet autoimmunity and type 1 diabetes in children – The DIPP birth cohort
Mattila M et al.

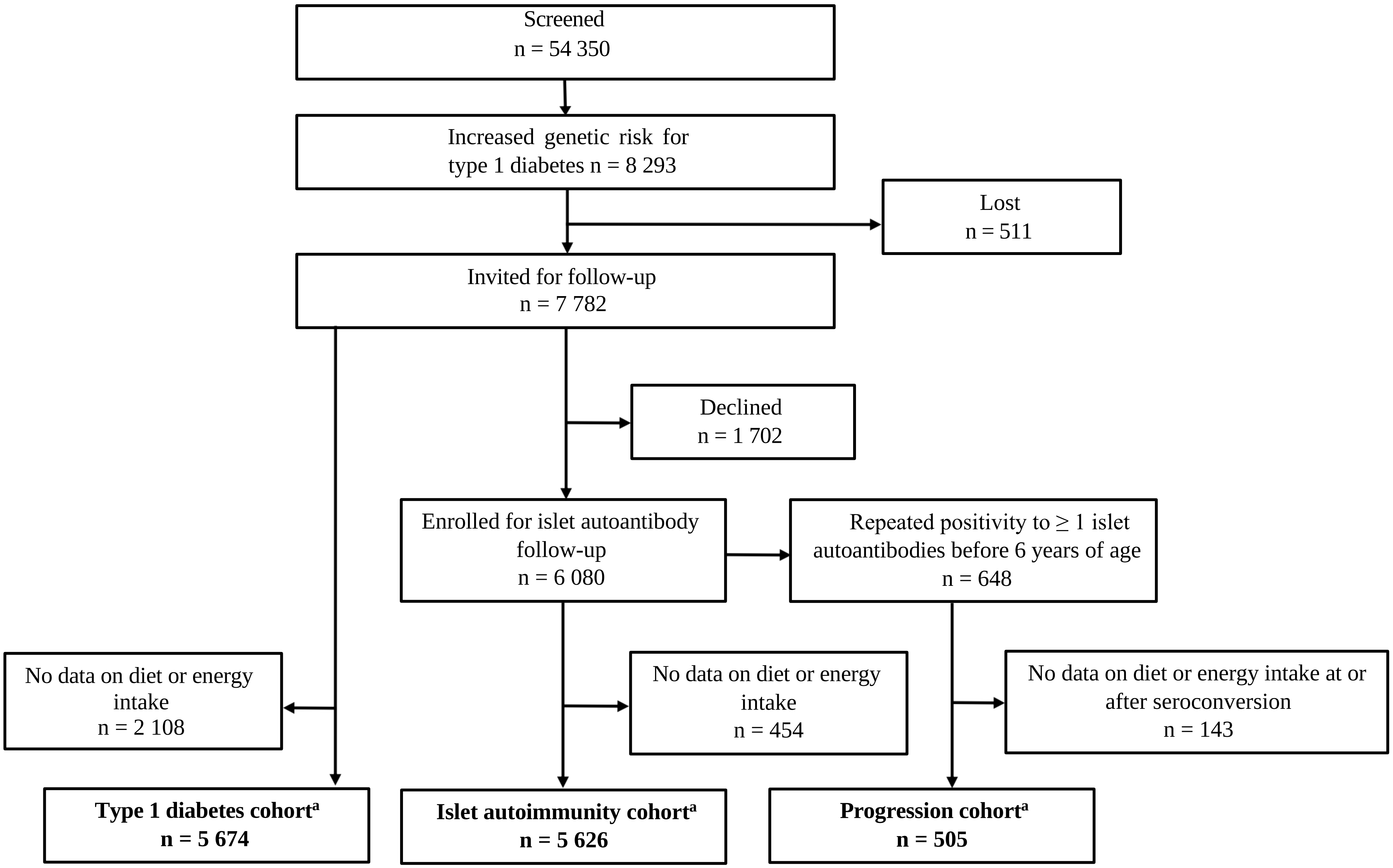

SUPPLEMENTARY FIGURE 1 DIPP Study participant flow chart

[a] Islet autoimmunity cohort is within type 1 diabetes cohort. Progression cohort (children having at least one autoantibody repeatedly positive) is within islet autoimmunity cohort
