# Supplementary material for: Fruit, berry, and vegetable consumption and the risk of islet autoimmunity and type 1 diabetes in children—the Type 1 Diabetes Prediction and Prevention birth cohort study
Source: Am J Clin Nutr. 2023 Dec 23;119(2):537–45. doi: 10.1016/j.ajcnut.2023.12.014 (PMC10884602; doi:10.1016/j.ajcnut.2023.12.014)
Supplement: Multimedia component2 [file mmc2.docx]

SUPPLEMENTARY TABLE 1 Associations of consumption of fresh/unprocessed fruits, berries, and vegetables and the risk of islet autoimmunity, type 1 diabetes, and progression to type 1 diabetes from joint modelling

|  | **Islet autoimmunity** |  |  |  |  | **Type 1 diabetes** |  |  | **Progression to type 1 diabetes** | |  |
| --- | --- | --- | --- | --- | --- | --- | --- | --- | --- | --- | --- |
|  | **HR (95% CI)^a^**  **n=5626 (cases, n=247)** | **p-value** | **Energy adjusted**  **HR (95% CI)^b^**  **n=5626 (cases, n=247)** | **p-value** |  | **Energy adjusted**  **HR (95% CI)^b^**  **n=5674 (cases, n=94)** | **p-value** |  | **Energy adjusted**  **HR (95% CI)^c^**  **n=505 (cases, n=64)** | **p-value** | |
| Fruits (without berries) | 1.05 (1.00, 1.08) | 0.032 | 1.03 (1.01, 1.05) | 0.002 |  | 1.05 (1.02, 1.08) | 0.001 |  | 1.06 (1.01, 1.11) | 0.015 | |
| Apple fruits | 1.04 (0.96, 1.12) | 0.321 | 1.03 (0.99, 1.07) | 0.118 |  | 1.06 (1.00, 1.12) | 0.043 |  | 1.13 (1.02, 1.23) | 0.018 | |
| Banana | 1.12 (1.03, 1.22) | 0.008 | 1.07 (1.02, 1.11) | 0.004 |  | 1.10 (1.00, 1.19) | 0.040 |  | 1.07 (0.97, 1.17) | 0.162 | |
| Berries | 0.22 (0.09, 0.93) | 0.016 | 0.96 (0.88, 1.03) | 0.388 |  | ^d^ |  |  | 0.89 (0.67, 1.08) | 0.332 | |
| Fruit vegetables | 1.03 (0.94, 1.10) | 0.493 | 1.01 (0.96, 1.05) | 0.724 |  | 1.04 (0.98, 1.09) | 0.138 |  | 1.06 (0.99, 1.13) | 0.097 | |
| Root vegetables | 0.58 (0.15, 1.09) | 0.149 | 0.94 (0.75, 1.10) | 0.569 |  | 0.52 (0.29, 1.02) | 0.064 |  | ^d^ |  | |
| Leafy vegetables | 0.80 (0.22, 1.98) | 0.796 | ^d^ |  |  | ^d^ |  |  | 0.20 (0.01, 2.13) | 0.295 | |
| Cruciferous vegetables | 0.94 (0.31, 1.76) | 0.980 | 0.87 (0.35, 1.31) | 0.880 |  | 1.05 (0.53, 1.50) | 0.702 |  | 1.09 (0.29, 2.24) | 0.693 | |

^a^ Hazard ratios (HR) and credible intervals (CIs) per consumption of 10 grams of food item. Adjusted for sex, HLA genotype, and family history of diabetes of any type

^b^ HRs and CIs per consumption of 1 gram/MJ of food item. Adjusted for sex, HLA genotype, family history of diabetes of any type, and total energy intake

^c^ HRs and CIs per consumption of 1 gram/MJ of food item. Adjusted for, energy intake, sex, HLA genotype, family history of diabetes of any type, and age at seroconversion

^d^ Not assessed due to convergence problems
